# Spinal Cord Repair: From Cells and Tissue Engineering to Extracellular Vesicles

**DOI:** 10.3390/cells10081872

**Published:** 2021-07-23

**Authors:** Shaowei Guo, Idan Redenski, Shulamit Levenberg

**Affiliations:** 1 The First Affiliated Hospital, Shantou University Medical College, Shantou 515041, China; 2 Department of Biomedical Engineering, Technion—Israel Institute of Technology, Haifa 32000, Israel; idan.redenski@gmail.com

**Keywords:** spinal cord injury, extracellular vesicles, exosomes, tissue engineering, scaffolds, cells, functional recovery

## Abstract

Spinal cord injury (SCI) is a debilitating condition, often leading to severe motor, sensory, or autonomic nervous dysfunction. As the holy grail of regenerative medicine, promoting spinal cord tissue regeneration and functional recovery are the fundamental goals. Yet, effective regeneration of injured spinal cord tissues and promotion of functional recovery remain unmet clinical challenges, largely due to the complex pathophysiology of the condition. The transplantation of various cells, either alone or in combination with three-dimensional matrices, has been intensively investigated in preclinical SCI models and clinical trials, holding translational promise. More recently, a new paradigm shift has emerged from cell therapy towards extracellular vesicles as an exciting “cell-free” therapeutic modality. The current review recapitulates recent advances, challenges, and future perspectives of cell-based spinal cord tissue engineering and regeneration strategies.

## 1. Introduction

Spinal cord injury (SCI) refers to damage to the spinal cord that temporarily or permanently changes its function. Worldwide, the average prevalence of traumatic and non-traumatic SCI is estimated to be 1:1000, and the annual incidence ranges from 2.1 to 130.7/million in developing countries and from 15 to 39/million in industrialized countries [1]. An estimated 3 million people live with traumatic SCI globally, with about 180,000 new cases reported every year [2]. SCI often leads to devastating long-lasting neurological deficits manifested by dysfunctions of the motor, sensory or autonomic nervous system below the level of injury. It also brings tremendous psychological, social and economic burdens. The most common causes of traumatic SCI are traffic accidents, falls and violence, while degenerative cervical myelopathy is the primary cause of non-traumatic SCI [3]. Current therapeutic strategies include surgical decompression, therapeutic hypothermia, and pharmacological agents such as methylprednisolone, GM-1 ganglioside, minocycline, riluzole and gacyclidine, as have been reviewed in detail elsewhere [4]. These neuroprotective and regenerative interventions are expected to improve long-term neurological and functional outcomes, yet the success in inducing substantial functional recovery remains limited.

Neurological outcome after SCI is determined by its pathophysiology, which is associated with a complex and dynamic set of cellular and molecular events. The initial mechanical insult injures neurons and non-neuronal cells and disrupts spinal vasculature and the blood–spinal cord barrier. This is followed by a cascade of secondary events, which further damages the spinal cord tissue and renders the microenvironment non-permissive for regeneration. In the acute phase (2 to 48 h), a series of events take place, including free-radical formation, neurotransmitter accumulation (excitotoxicity), calcium influx, ionic imbalance and lipid peroxidation [5]. Intraparenchymal hemorrhage increases the level of pro-inflammatory cytokines and recruits inflammatory cells, such as macrophages, neutrophils and lymphocytes, into the spinal cord. The profound inflammatory milieu progressively adds to spinal cord swelling and worsens the injury [6]. Meanwhile, the impaired autoregulatory capacity of the injured vasculature, coupled with hypovolemia and hemodynamic shock, results in compromised spinal cord perfusion and ischemia [5,6]. Prolonged ischemia propagates neuronal death. In the subacute phase (2 days to 2 weeks), further ischemia occurs due to ongoing edema, vasospasm and thrombosis [6]. It also involves apoptosis, demyelination, Wallerian degeneration, axonal dieback and cyst formation [5]. Importantly, in response to the inflicted injury, astrocytes proliferate in a process called reactive gliosis. Hypertrophic astrocytes tightly interweave their long processes, forming a tenacious, mesh-like, growth-blocking array [7]. In addition to serving as a physical barrier to halt the advancement of regenerating axons, the scar tissue acts as a chemical barrier by secreting inhibitory molecules such as chondroitin sulfate proteoglycans (CSPGs) [7]. The drastic upregulation of CSPG expression causes the collapse of axonal growth cones and inhibits the migration and differentiation of oligodendrocyte progenitor cells [8]. On the other hand, the glial scar and scar-forming astrocytes play beneficial roles. They modulate the balance of inflammatory activities, serve as a restrictive border to limit fibrotic tissues and macrophages, and form bridges for axonal regrowth [9,10,11,12]. The ablation of glial scars in the acute or subacute phase leads to lesion expansion and worse recovery [13]. As the injury progresses into the intermediate (2 weeks to 6 months) and chronic (over 6 months) phases, the glial scar matures and cystic cavities coalesce [6].

**Figure 1:**
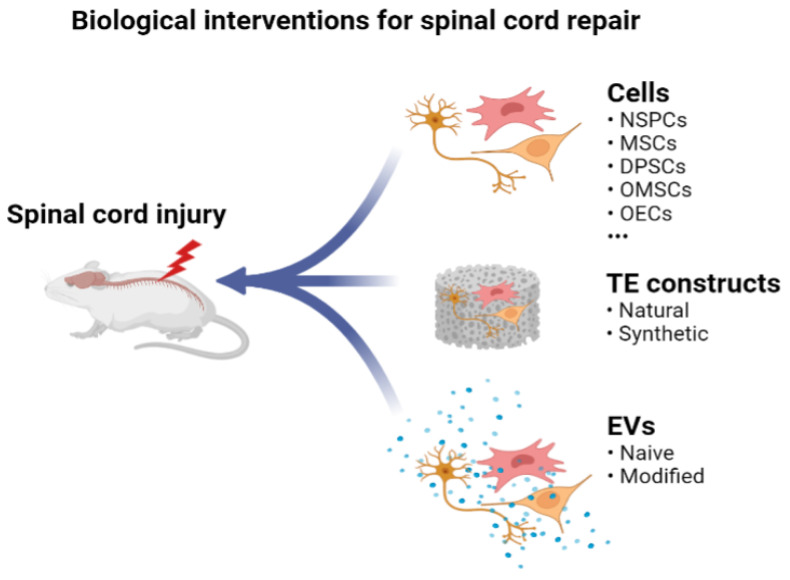
Illustration of biological interventions for spinal cord repair. Cells, tissue-engineered (TE) constructs and extracellular vesicles bear promising therapeutic potentials, and they can be combined for repairing damaged spinal cord tissues. The figure illustration was created using BioRender (https://biorender.com/, accessed on 26 June 2021).

In recent years, cell-based therapies have been immensely implemented to address multifaceted pathophysiological processes, aiming to promote neuroprotection, immunomodulation, axon regeneration, neuronal relay formation and myelin regeneration [14]. In this review, we summarize promising therapeutic potentials of neural stem/progenitor cells (NSPCs), mesenchymal stem/stromal cells (MSCs), dental pulp stem cells (DPSCs), oral mucosa stem cells (OMSCs) and olfactory ensheathing cells (OECs), as well as cell-based tissue engineering approaches and the use of extracellular vesicles to promote SCI repair and functional recovery (Figure 1).

## 2. Cell-Based Therapies for SCI

### 2.1. Neural Stem/Progenitor Cells

**Figure 2:**
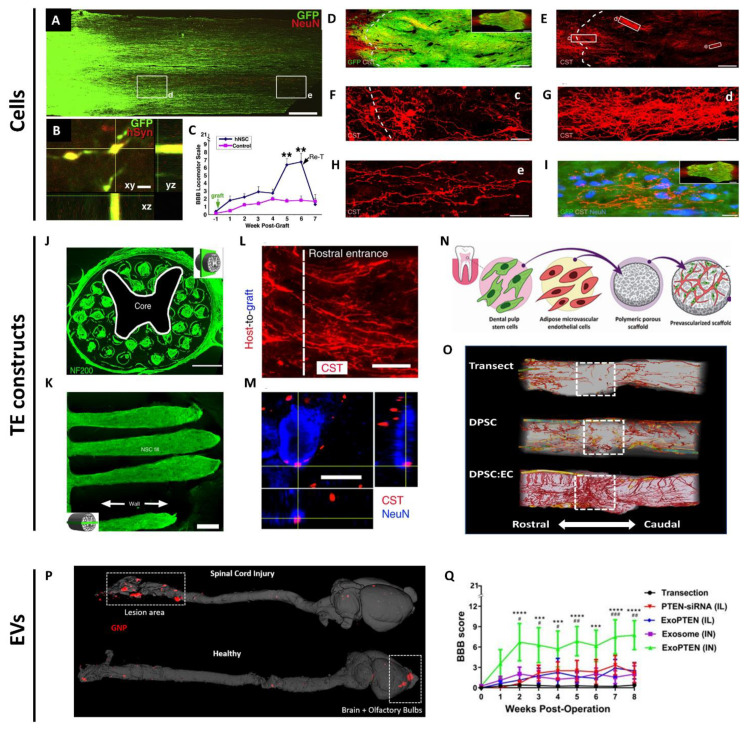
Examples of biological interventions using cells, tissue-engineered constructs or extracellular vesicles for spinal cord repair. (**A**) GFP-labeled human neurons robustly extend axons into the host spinal cord caudal and rostral to the complete T3 transection site (caudal shown). (d) and (e) demonstrate axonal extensions from human neurons close to and far away from the engrafted site. (**B**) GFP-labeled human axon terminals express human-specific synaptophysin (red) in host gray matter. (**C**) Functional recovery after human NSC grafts to sites of T3 complete transection, but re-transection at rostral interface of graft abolished the effect. ** *p* < 0.01. Reproduced with permission from Lu et al. [17]. Copyright 2012 Elsevier. (**D**–**I**) Corticospinal tract (CST) axons (red) robustly regenerate into GFP-expressing NPC grafts and surround neurons in the center of the graft in the site of complete T3 transection site. (c–e) in (**F**–**H**) represent higher magnifications of corresponding rectangles from (**E**) Reproduced with permission from Kadoya et al. [18]. Copyright 2016 Springer Nature. (**J**) A cross-section through an implanted biomimetic 3D-printed scaffold labeled for axons (NF200). (**K**) The scaffold channels are structurally intact and filled with GFP-expressing NSCs 6 months post transplantation. (**L**,**M**) CST axons (red) penetrate the scaffold, extend linearly in a caudal direction, and come into contact with NeuN-labeled neurons (blue) inside the channel. Reproduced with permission from Koffler et al. [33]. Copyright 2019 Springer Nature. (**N**) Schematic illustration of fabricating prevascularized tissues by co-culturing DPSCs and endothelial cells on PLLA/PLGA scaffolds. (**O**) 3D reconstruction of vasculature (red) spanning the lesion segment (dashed boxes) in transection control, DPSC-scaffolds, or prevascularized scaffold groups 8 weeks post-treatment. Reproduced with permission from Guo et al. [34]. Copyright 2020 John Wiley and Sons. (**P**) Micro-CT scanning of the CNS tissues in the injured (upper panel) and healthy rats (lower panel), after intranasal administration of gold nanoparticle (GNP)-labeled exosomes (red). (**Q**) Weekly locomotor scores of SCI rats left untreated, or treated with intralesional PTEN-siRNA or ExoPTEN, intranasal exosome (IN) or ExoPTEN. # *p* < 0.05, ## *p* < 0.01, ### *p* < 0.001 between exosome (IN) and ExoPTEN (IN). *** *p* < 0.001, **** *p* < 0.0001 between transection control and ExoPTEN (IN). Reproduced with permission from Guo et al. [35]. Copyright 2019 American Chemical Society. Scale bars, 600 μm (**C**); 3 μm (**B**); 240 μm (**D**,**E**); 60 μm (**F**); 30 μm (**G**); 20 μm (**H**,**I**); 500 μm (**J**); 250 μm (**K**); 10 μm (**L**); 5 μm (**M**).

NSPCs are multipotent progenitors isolated from the CNS, typically grown as neurospheres with the capacity to differentiate into neurons and glia. NSPCs have high therapeutic potential for reconstructing the spinal cord lesion, as they can potentially form relay circuits to bridge functional connectivity between injured spinal cord segments. Host axons can penetrate and connect with the engrafted NSPCs. Reciprocally, the transplanted NSPCs can grow, extend their axons, and form synapses with host axons [15]. The Fischer laboratory provided the first demonstration that transplanted NSCs were able to extend long distances in the injured spinal cord, form connections with descending motor pathways and improve bladder and motor functions [16]. However, the SCI was only partial, so the efficacy in a more severe model remained to be examined. In a subsequent study, Tuszynski et al. engrafted green fluorescent protein (GFP)-expressing NSCs with a cocktail of ten trophic factors two weeks after complete SCI in rats. Long-distance growth and connectivity of NSCs with the host axons and modest motor recovery were achieved (Figure 2A–C) [17]. This phenomenon was neither cell-specific nor species-specific, thus bearing great translational potential. In a later study, the same group achieved a key milestone by discovering that engrafted caudalized and homotypic NPCs induced robust corticospinal axons into the grafts to form functional synapses with grafted neurons, which restored skilled forelimb function in a cervical SCI model in rats (Figure 2D–I) [18]. In a more clinically relevant study, the group engrafted human NPCs into a primate model of cervical SCI. The grafts survived up to nine months post-implantation, expressed neuronal and glial markers, extended thousands of long axons and formed synapses with host motor neurons and improved forelimb function [19]. This study resonates an earlier report that human spinal cord-derived NPCs reliably enhanced the functional outcome after SCI when transplanted acutely or sub-acutely [20]. While NSPC transplantation in the subacute phase resulted in functional recovery, such benefits were limited in the chronic phase. Modification of the microenvironment of the injured spinal cord focusing on glial scar formation and inflammatory phenotype should be considered to maximize the therapeutic potential of NSPCs in the chronic phase [21]. Another translational concern is the risk of immune rejection, and therefore the use of immunosuppressants, since NSPCs are most commonly derived from embryonic stem cells or allogeneic adult sources [22], and human NSPCs with time in culture will increase their Major Histocompatibility Complex Class I and II expressions [23]. The induced pluripotent stem cell (iPSC)-derived and directly reprogrammed NSCs have offered the possibility of autologous transplantation, which mitigates immune rejection concerns [24,25]. However, even for autologous iPSC transplantation, there is a chance of triggering an immune response. Therefore, the immunogenicity and genetic stability of iPSC-derived NSPCs should be evaluated before their clinical applications [26,27]. Recently, some of the works using NSPCs for SCI repair have been placed in the focus of National Institutes of Health-supported investigations to support independent replication. These investigations highlighted important issues relating to the potential application of NSC transplantation for severe SCI, including a need to further refine transplantation techniques [28,29] and follow other practices to reduce the lack of rigor [30,31,32].

### 2.2. Mesenchymal Stem/Stromal Cells

The use of MSCs represents one of the most promising strategies in regenerative medicine. According to the International Society for Cellular Therapy, the following criteria must be met to define multipotent MSCs: (1) plastic-adherent when maintained in standard culture conditions using tissue culture flasks; (2) ≥ 95% of the population must express CD105, CD73 and CD90, and must lack expression (≤2% positive) of CD45, CD34, CD14 or CD11b, CD79a or CD19 and HLA class II as measured by flow cytometry; and (3) must be able to differentiate in vitro into osteoblasts, adipocytes and chondroblasts [36]. MSCs have been isolated and characterized from various sources, such as bone marrow, adipose tissue, umbilical cord, placenta, amniotic fluid, dermis, synovial fluid and periosteum [37]. The majority of MSCs used in SCI clinical trials are derived from bone marrow. MSCs are extremely attractive in the landscape of translational medicine, as reflected by their dominance in registered clinical trials for SCI patients, largely due to overwhelming evidence of their regenerative effects in preclinical studies, ease of isolation and preservation, rapid proliferation, limited ethical concerns and, very importantly, their safety profile [38]. From another aspect, MSCs are appealing because of their “homing” property, i.e., being able to preferentially migrate to the damaged tissues, mediated by inflammatory or chemotactic factors and the SDF1-α/CXCR4 axis [39,40,41]. MSCs exert their therapeutic effects via multiple paracrine mechanisms, including anti-inflammation, immunomodulation, neuroprotection, and pro-angiogenesis [18]. The conditioned medium from MSCs can be used to promote functional recovery after SCI, suggesting that cell transplantation may not be needed to attain functional outcomes [42]. Collectively, these paracrine capacities render MSCs promising in mitigating secondary tissue damage after SCI. Despite encouraging preclinical findings, substantial clinical trials applying MSC therapy for CNS injuries fall short of expectations. Many factors affect the heterogeneity and, ultimately, the clinical outcome of MSCs. For instance, during the preparation of MSCs, donor variations (e.g., health status, genetics, gender and age), different sources of isolation (e.g., bone marrow, umbilical cord and adipose tissue) and various methods of isolating cells (needle vs. biopsy, enzymatic vs. mechanical dissociation) could result in heterogeneity of the MSC products. In addition, the administration route, injection site, infusion time, and cell carrier materials can affect the residence time, viability, and migratory capacity of MSCs, making the therapeutic outcomes unpredictable [43]. Standardized manufacturing processes and potential bioengineering approaches should be introduced to develop more potent and predictable MSC-based therapies.

### 2.3. Dental Pulp Stem Cells

DPSCs are a source of easily accessible self-renewal stem cells that reside in the dental pulp cavity [44]. They originate from migrating neural crest cells and express both mesenchymal markers, such as CD73, CD90 and CD105, and neural lineage markers, such as nestin, βIII-tubulin, glial fibrillary acid protein and neuronal nuclei [45,46]. This versatile cell population is readily harvestable from wisdom teeth, which are considered medical waste. Apart from their simplicity, lack of ethical controversy and convenience of isolation, under defined culture conditions, they can differentiate into odontoblastic, osteoblastic or neurogenic lineages [47,48,49]. The regenerative potential of DPSCs was established for pulpal and bone tissue regeneration, and they exhibited the ability to induce cardiac tissue regeneration, comparable with that of MSCs [50,51,52,53]. DPSCs bear promising tissue regeneration implications for clinical utility, particularly attractive for treating CNS disorders [54,55]. Due to their neural crest origin, DPSCs possess substantial neurotrophic effects able to secrete neurotrophic factors to promote neuronal survival, differentiation and migration [56,57]. Moreover, they could facilitate the regeneration of transected axons and induce functional recovery in SCI by multiple neuroregenerative mechanisms, namely, coupling of angiogenesis and neurogenesis [34], inhibiting the apoptosis of neural cells, antagonizing multiple axon growth inhibitors and replacing lost cells by differentiating into mature oligodendrocytes [54]. However, clinical trials are just underway to evaluate the safety and feasibility of autologous DPSCs in patients with chronic disability after stroke [58], or the efficacy and safety of allogeneic DPSCs in patients with acute stroke (NCT04608838). While these studies would offer some information about the safety and efficacy of DPSC therapy for stroke patients, similar investigations should be initiated on SCI patients.

### 2.4. Oral Mucosa Stem Cells

Wounds within the oral mucosa that lines the oral cavity heals rapidly with minimal scar formation. This preferential healing phenomenon led to the investigation and report of a progenitor population within the oral mucosa lamina propria (OMLP) [59]. It was characterized that the neural crest-derived OMLP harbors a novel stem cell population, termed OMSCs [60]. Immunophenotyping revealed a primitive neural crest (NC) stem cell phenotype, with the expression of the pluripotency transcription factors Oct4, Nanog and Sox2, the NC markers nestin, Snail and p75 and the neuronal marker βIII tubulin. Regardless of donor age, a small biopsy of the oral mucosa can generate trillions of OMSCs, which can differentiate into mesoderm, endoderm or ectoderm lineages [60]. Transplantation of OMSCs ameliorated SCI-induced neurologic bladder symptoms by inhibiting apoptosis and suppressing neuronal activation in the neuronal voiding centers [61]. Forced upregulation of neurotrophic factor expression of OMSCs via a medium-based differentiation protocol provides neuroprotection in sciatic nerve injury and Parkinson’s models [62,63]. These differentiated OMSCs, when combined with 3D matrices, could promote functional recovery in rats with complete spinal cord injury [64]. The potential for the clinical applications of OMSCs to restore damaged spinal cord tissues is attractive, but appropriate clinical trials are needed to confirm their regenerative capability.

### 2.5. Olfactory Ensheathing Cells

Over the last three decades, autologous olfactory tissues have been one of the most promising sources for SCI repair. The regenerative potential was ascribed to OECs, the glial cells found exclusively in the olfactory tissues. As the name indicates, OECs ensheathe primary olfactory axons from the lamina propria of the olfactory mucosa (OM) en route to the olfactory bulbs (OB) [65]. Whenever an injury occurs in life, the olfactory receptor neurons from mucosa epithelium die back, but OECs encourage them to regenerate and re-synapse with neurons in the OB [65]. OECs’ regenerative capacities are manifested by expressing neurotrophic factors, phagocytosing cellular debris, secreting ECM molecules for bridging newly regenerated axons, attenuating neuroinflammation, and promoting angiogenesis [66]. There are differences in cellular compositions and therapeutic efficacies between cell biopsies from OM and OB. The OM contains more cell types, including OECs, MSCs, fibroblasts, Schwann cells and resident macrophages, while the OB has fewer cell types [66]. OECs isolated from OM are more beneficial for regeneration purposes, with higher secretion of neurotrophic factors, such as neurotrophin-3, nerve growth factor and brain-derived neurotrophic factors, increased cavity prevention and axonal regeneration in rat SCI models [66]. From the surgical health perspective, OEC isolation from OM is more advantageous, as isolation from the OB requires major intracranial operation and poses a risk of developing anosmia after surgery [66]. Although neurological improvements have been achieved following OEC transplantation in animal and human SCI models, therapeutic outcomes are inconsistent. Discrepancies in outcomes are largely due to differences in OEC purity. In fact, mixed suspensions of olfactory cells containing OECs and fibroblasts [67,68], or whole pieces of olfactory mucosal tissues [69,70], have been grafted into SCI patients, but no purification or cellular composition analysis was described. The variations in cell preparations not only make it very difficult to compare outcomes of OEC transplantation studies, but also pose a risk to patient safety. Dlouhy et al. reported a case of an olfactory mucosa cell transplant recipient who developed an intramedullary multicystic mass containing large amount of thick mucus-like material at the site of implantation eight years after surgical implantation to treat her paralysis [71]. This highlights the necessity to identify and purify olfactory cell populations before transplantation [66].

## 3. Tissue-Engineered Constructs for SCI

### 3.1. Requirements of Biomaterials for SCI Repair

Recent years have witnessed rapid advances in tissue engineering and material science, particularly in the development and application of biomaterial scaffolds for SCI repair. Scaffolds can serve as bridges to connect the separated spinal cord segments and as platforms for stem cell attachment, differentiation, integration, or for incorporating and releasing bioactive factors [72]. When choosing and designing scaffolds for SCI repair, several aspects need to be taken into consideration. (1) Biocompatibility. The breakdown of scaffolds should be non-toxic and not elicit an immune response [73]. (2) Biodegradability. Scaffolds should temporarily support axonal regeneration and degrade over time [72]. Maintenance of scaffold structure for over four weeks is important to support axonal growth and organization across the lesion site [33]. (3) Mechanical property. Scaffolds should not only withstand forces generated from the spine and the surrounding tissues [72], but also match the Young’s modulus (100 Pa to 10 kPa) of the soft spinal cord tissue [74]. (4) Architecture. Scaffolds need to be porous to enable cell attachment, migration and nutrient permeability [75]. In addition, scaffolds should be fabricated to be more personalized such that they would fit precisely into the defect and their internal geometries mimic the anatomy of the spinal cord. This will offer topographical cues to guide regenerating axons to form correct synapses. Three-dimensional printing allows scaffold fabrication to be more precise and controllable. Recently, Koffler et al. printed 3D biomimetic hydrogel scaffolds tailored to the dimensions of rats’ spinal cords. Those scaffolds, when loaded with NPCs, supported host axon regeneration, synaptic transmission, and functional recovery in a complete transection model (Figure 2J–M) [33].

Broadly, scaffolds are divided into natural and synthetic types. Natural materials are generally nontoxic, biocompatible, biodegradable, highly porous and more natural for cellular interactions. However, some natural materials may elicit undesired inflammatory responses [76]. Synthetic materials are more advantageous in terms of low inflammatory response, tunable biodegradability, customized porosity, and controllable mechanical properties [77]. Several of the most commonly used natural and synthetic scaffolds for SCI repair are reviewed here.

### 3.2. Natural

#### 3.2.1. Collagen Scaffolds

Collagen is the most abundant protein of the extracellular matrix in our body. It is biocompatible and biodegradable, with low antigenicity. Moreover, its fibrous structure is beneficial for the adhesion of cells and bioactive molecules [76]. The Dai laboratory has long been working on functional collagen scaffolds to tune endogenous NSCs for complete SCI repair. In response to injury, endogenous NSCs become activated and migrate to the rostral and caudal lesion stumps, but only a few of them migrate into the center [78]. Even if those activated NSCs do reach the center, they do not give rise to neurons, but astrocytes and oligodendrocytes [79]. To enhance the recruitment of endogenous NSCs, the group designed radially aligned electrospun collagen/PCL scaffolds with a continuous gradient of collagen-binding domain (CBD)-stromal cell-derived factor-1α (SDF1α). The CBD-SDF1α gradient scaffolds successfully manipulated NSCs’ migratory behavior [80]. Building on this knowledge, human umbilical cord-derived mesenchymal stem cells (hMSCs) or human fetal spinal cord-derived neural stem cells (hNSCs) were seeded on longitudinal collagen sponge scaffolds and implanted to rats with completely transected SCI. While both types of 3D-engineered constructs effectively reduced glial scar formation, more regenerating neurons and nerve fibers were found in the lesion after hNSC-scaffold implantation [81]. The collagen scaffolds, termed NeuroRegen scaffolds, were loaded with MSCs or NSCs for implantation in chronic SCI patients (ASIA grade A), and functional impairment scale electrophysiological outcomes were expected to improve (NCT02688049). Taken together, collagen scaffolds are a promising platform for modulating the injury-activated NSCs and loading stem cells for SCI repair.

#### 3.2.2. Chitosan Scaffolds

Chitosan is a naturally available polysaccharide composed of *N*-glucosamine and *N*-acetyl-glucosamine [82]. Chitosan scaffolds can diminish fibrous glial scarring, modulate the inflammatory response, promote reconstitution of spinal cord tissue, neovascularization and axonal growth and, importantly, lead to long-lasting gain in the locomotor functional recovery in a bilateral dorsal hemisection model [83]. In another study, Li et al. further incorporated neurotrophin-3 (NT3) into chitosan scaffolds and implanted them into a 5 mm gap of fully transected rat spinal cord. The NT3-coupled chitosan scaffolds provided an optimal microenvironment for the robust activation of endogenous NSCs and attracted those NSCs to the lesion center to differentiate into neurons. The differentiated neurons formed nascent circuits with the descending and ascending tracts, enabling beneficial neurological outcomes [84]. In a subsequent study, the group tested the regenerative potential of NT3-chitosan scaffolds in rhesus monkeys which have similar genetics and physiology to humans. The NT3-loaded chitosan scaffolds elicited robust de novo neural regeneration, featuring long-distance axonal growth of the corticospinal tract over a 1 cm lesion area and functional recovery in a T8 hemisection model, moving one step closer to clinical trials [85]. In addition, the chitosan-based hydrogel was used to encapsulate bone marrow MSCs, and support their paracrine activity in the SCI site [86].

#### 3.2.3. Hyaluronic Acid Scaffolds

Hyaluronic acid (HA) is a large natural polysaccharide consisting of two disaccharide units, including *N*-acetyl-glucosamide and d-glucuronic acid [82]. HA has gained considerable attention as a scaffolding biomaterial for repairing CNS injuries as it is widely found in the CNS and highly biocompatible. HA hydrogels possess an interconnected porous structure, which allows for nutrient transport and cell penetration [87]. Moreover, HA hydrogels could reduce glial scar formation via inhibiting the migration, chemotaxis and proliferation of lymphocytes [88]. It is important to note that the molecular weight and modulus of HA hydrogels affect the anti-scarring and NPC differentiation [87]. HA can be blended with regenerative cells, neurotrophic factors, cell adhesion molecules or bioactive compounds to achieve better functional outcomes [87].

### 3.3. Synthetic

#### 3.3.1. PLA/PLGA Scaffolds

Poly-lactic acid (PLA) is a polymer of lactic acid with good biodegradability. Due to their safety profiles, the Food and Drug Administration (FDA) has approved several PLA formulations for medical applications [76]. PLA scaffolds can be fabricated with different forms for SCI repair, such as hydrogels, microporous sponges with inner channels, microfibers and nanofibers [82]. PLA scaffolds with aligned microfibers can foster robust regeneration of vascularized spinal cord tissue with long-distance, rostrocaudal axons [89]. Freeze-dried PLA microporous guidance scaffolds loaded with brain-derived neurotrophic factors are well tolerated in vivo and can promote the growth of axons and vessels [90]. PLA scaffolds seeded with Schwann cells genetically modified to produce bi-functional neurotrophins effectively promote axonal regeneration in rats with complete spinal cord transection [91].

Poly (lactic-co-glycolic acid) (PLGA) is an FDA-approved synthetic biocompatible and biodegradable co-polymer of polylactic acid and polyglycolic acid [82]. PLGA is a flexible material with tunable degradation profiles by adjusting its lactide and glycolide ratio [76]. PLGA, in the forms of hydrogels, microspheres or nanoparticles, has been tested in SCI models to achieve functional recovery [82]. PLGA-based scaffolds also act as vehicles for delivering cells and therapeutic factors. Teng et al. fabricated PLGA scaffolds with a degradation rate of about 30–60 days. The scaffolds were populated with NSCs and implanted into the rat hemisection model, promoting long-term functional recovery. The scaffolds themselves were found to impede scarring and subsequent cyst formation [92]. Similarly, the group seeded another cell type, MSCs, into the same scaffolds, and demonstrated that the scaffolds maintained MSC stemness and engraftment and promoted functional recovery [93]. Of note are the INSPIRE clinical trials (NCT02138110; NCT03762655) steered by InVivo Therapeutics, utilizing Neuro-Spinal scaffolds which combine PLGA and poly-l-lysine to examine the probable benefits for the treatment of thoracic SCI individuals (ASIA grade A) following implantation.

PLGA can be combined with poly-l-lactic acid (PLLA) to form the PLLA/PLGA scaffolds, which serve as biocompatible and biodegradable matrices for cell attachment, proliferation, differentiation and organization in muscle, bone and spinal cord tissue engineering [34,64,94,95,96]. Using the salt-leaching technique, highly porous PLLA/PLGA scaffolds can be fabricated with pore sizes of 212–600 µm and 93% porosity [34,64,94,95,96]. The implantation of such PLLA/PLGA scaffolds embedded with differentiated OMSCs induced endogenous repair in a complete transection model via the secretion of neuroprotective, immunomodulatory and axonal elongation-associated factors [64]. PLLA/PLGA scaffolds can also be prevascularized by a co-culture of DPSCs and endothelial cells. These prevascularized PLLA/PLGA scaffolds bear angiogenic and neurotrophic potentials capable of promoting revascularization, axon regeneration, myelin deposition and sensory recovery in the same transection model (Figure 2N,O) [34]. Furthermore, recently, a unique fabrication technique based on 3D printing was developed to generate highly oriented and anatomically personalized PLLA/PLGA scaffolds to match the aligned topography of spinal cord tissues. The oriented PLLA/PLGA scaffolds guided regenerating axons to linear conformations and supported the growth and guidance of iPSC-derived neurons in the complete spinal cord transection model [96].

#### 3.3.2. PCL Scaffolds

Poly-ε-caprolactone (PCL) is a biocompatible and biodegradable aliphatic polyester, widely used in tissue engineering [76]. In one study, salt-leached porous PCL scaffolds were fabricated with five different macro-architectures, namely, cylinder, tube, channel, open-path with core and open-path without core. The open-path designs provided contact guidance, allowed myelinated axons to extend across the entire defect length and enhanced spinal cord regeneration. In another study, a modified salt-leaching technique was used, which improved scaffold porosity up to 60% and reduced the elastic modulus. Linear growth of axons, minimum scar tissue formation and scaffold maintenance over 4 weeks were observed in a rat T3 full-transection model [97]. Electrospinning can also be used to fabricate PCL scaffolds with loop mesh and biaxial aligned microscale topographies for inducing the neuronal differentiation of stem cells and guiding the regenerating axons [98]. PCL scaffolds can be combined with NT3, self-assembling peptides and stem cells for the repair of SCI [76].

## 4. Extracellular Vesicles for SCI Repair

### 4.1. The Paradigm Shift

In theory, cell therapy helps to repair damaged tissues via direct cell replacement. Indeed, numerous preclinical and clinical studies have proved the efficacies of transplanted cells, and high-resolution in vivo imaging methods readily track and reveal cell migration to the site of injuries [99]. Some engrafted cells could differentiate to replace lost cells in the lesion [54]. However, relatively few cells were able to engraft at the site of injuries, and most intravenously administered cells were caught in lung capillaries or cleared. In addition, the conditioned medium of cells produced similar therapeutic effects to the delivery of the cells [100]. Therefore, once anticipated to function in direct cell replacement for damaged tissues, it is now widely established that paracrine effects recapitulate to a large extent the therapeutic effects of parental cells.

Most cells secrete extracellular vesicles (EVs), lipid membrane-enclosed nanovesicles that contain growth factors, signaling lipids, mRNAs and regulatory miRNAs to facilitate intercellular communications under both physiological and pathological conditions [101]. EVs comprise a heterogeneous population of membranous vesicles of various origins, including exosomes, microvesicles, microparticles, apoptotic bodies, ectosomes, oncosomes, and many others [102]. Classically, EVs are broadly divided into microvesicles, larger vesicles directly released from cells by budding of the cell membrane and exosomes, smaller vesicles secreted via multivesicular endosomal pathway [101].

### 4.2. EV Therapy for SCI

EVs were originally considered as “garbage bins” for removing unwanted substances from the cells [103]. However, ongoing advances in the field of EVs have made it increasingly clear that EVs have diverse functions and utilities, ranging from the maintenance of normal physiology to disease progression, from diagnostic and prognostic biomarkers to therapeutic targets and from skin rejuvenation to suppressing undruggable pancreatic cancer [101,104,105,106]. It is worth emphasizing that the ability of EVs to surmount the blood–brain barrier and gain access to the CNS has made their use tremendously promising in the treatment of neurological diseases [107]. Several preclinical studies demonstrated EVs’ potential to improve neurological outcomes in animal models of neurological diseases. For instance, human bone marrow MSCs-derived EVs could rescue pattern separation and spatial learning impairments in traumatic brain injury mice [108]. In a stroke model, EVs derived from rats’ MSCs enhanced neurite remodeling, neurovascular plasticity and improved functional recovery [109]. MSC-derived EVs could also ameliorate autistic-like behaviors of BTBR mice following intranasal administration [110]. Intranasal delivery of MSC-derived A1-exosomes, batches of MSC-derived EVs with robust anti-inflammatory properties, suppressed inflammation and prevented abnormal neurogenesis and memory dysfunction after status epilepticus [111]. Human teeth stem cell-derived EVs, given intranasally, could improve motor symptoms and normalize tyrosine hydroxylase expression in the striatum and substantia nigra in a Parkinson’s model. Interestingly, intranasal delivery appears to be an attractive method for gaining access to the CNS tissues. Similarly, intranasal administration of macrophage-derived EVs effectively reached the brain tissue in a mouse model of Parkinson’s disease, as revealed by optical imaging [112]. In an ischemic stroke model, in vivo CT imaging of gold nanoparticle-labeled EVs revealed that intranasal EV administration resulted in better EV accumulation in the lesion than intravenous injection [113]. The same group further demonstrated that intranasally delivered MSC-EVs selectively targeted brain pathologies in an array of neurodegenerative and neurodevelopmental disorders [114]. Although the mechanisms of intranasal EVs reaching the CNS tissues are not fully elucidated, transport across the epithelial cell layer directly to the circulation and transport along the olfactory nerve are the possible routes [112]. Apart from the migration and efficacy studies of EVs on brain pathological models, the use of EVs for treating spinal cord injuries has also gained considerable attention. EVs isolated from different cell sources and species readily demonstrated their efficacy in improving motor recovery across different injury models, mostly by ameliorating the non-permissive microenvironment (Table 1). Although several pre-clinical studies have focused on the EV-mediated transfer of certain proteins (e.g., vascular endothelial growth factor) or microRNAs (e.g., miR-133b or miR126) and showed neuroregeneration [43,115,116], the therapeutic actions and beneficial effects of EVs were most probably mediated by the transfer of a battery of molecules (growth factors, signaling lipids, mRNAs, regulatory miRNAs, etc.), rather than by one single molecule.

### 4.3. EVs as Drug Delivery Vehicles

In addition to their innate therapeutic potentials, EVs have also been exploited as advantageous drug delivery vehicles. The advantages include minimal or no cytotoxicity, a small size for penetration into deep tissues, capability to escape rapid clearance by the reticuloendothelial or mononuclear phagocyte system and ability to penetrate the blood–brain barrier [135]. Loading therapeutic cargos into EVs can be broadly divided into two major approaches: endogenous and exogenous loading. Endogenous loading refers to manipulations of parental cells to overexpress molecules of interest, such that secreted EVs contain those desired cargos. In contrast, exogenous loading involves post-isolation modifications of EVs. As of now, various methods have been developed to load bioactive components into EVs, including electroporation, sonication, extrusion, freeze and thaw cycles, incubation with membrane permeabilizers and co-incubation with modified cargos [101,135]. With these technologies, proteins, siRNAs, microRNAs and small molecule compounds. including doxorubicin, curcumin and paclitaxel, have been successfully loaded into EVs [101]. A robust, efficient, and reproducible method was demonstrated recently, using a co-incubation of hydrophobically modified small interfering RNAs and EVs. In addition, this method did not alter the EV size and integrity and was highly efficient in RNA interference in vitro and in vivo [136]. This opens exciting opportunities for developing innovative therapies for neurological disorders. We showed that in rats with complete SCI, intranasal delivery of MSC-derived exosomes could penetrate the blood-brain barrier, home selectively to the spinal cord lesion and show affinity to neurons within the lesion. When these exosomes were loaded with phosphatase and tensin homolog small interfering RNA, termed ExoPTEN, they migrated from the nose and silenced *PTEN* expression in the lesion. Furthermore, the loaded exosomes promoted robust axonal regeneration and angiogenesis, accompanied by decreased astrogliosis and microgliosis. Moreover, the intranasal ExoPTEN treatment partially restored electrophysiological and structural integrity and, most importantly, enabled remarkable functional recovery. This rapid, non-invasive approach, using cell-free nano-vesicles carrying molecules to target pathophysiological mechanisms, suggests a novel strategy for clinical translation to SCI and beyond (Figure 2P,Q) [35,137].

## 5. Challenges and Future Perspectives

Despite substantial efforts and progress that have been made in the field of SCI treatment by cell or cell-embedded biomaterial transplantation, significant challenges remain. Efforts are needed to improve graft survival and host regeneration, establish and maintain functional synaptic connections, identify the best neurons for improving connectivity, guide transplanted cells to appropriate targets and avoid maladaptive connectivity [138]. It is important to realize that enormous axon growth forcibly induced by cell grafts or neurotrophic cocktails is not always functionally beneficial. Regenerating axons can end abortively or form ectopic connections which could be detrimental to functional recovery [139]. To this end, neuromodulation strategies and rehabilitation could be employed to direct regenerating axons toward forming functional synapses with target neurons and facilitate clinically meaningful recovery after SCI [140]. Other important factors to consider include standardization, quality control, potency evaluation, scale-up, Good Manufacturing Practice (GMP), and logistics of cell therapies. When 3D biomaterials are used to deliver cells, several issues need to be taken into consideration before clinical translation, such as biodegradation rate, biocompatibility, material safety and hierarchical structures of the biomaterials. Lastly, while most preclinical cell transplantation studies are performed in the acute or subacute phase, chronic SCI remains understudied and is more of a challenge than acute or subacute SCI.

EV-based therapies also face many challenges toward clinical translation. One bottleneck lies in manufacturing scalable, clinical-grade EVs. Currently, EV isolation remains largely from parental cells cultured as monolayers on 2D plastic dishes. However, this culture condition is not physiologically relevant, and the production usually involves a large volume of medium, space and intensive labor, while the yield of EVs is limited. To overcome this limitation, we recently engineered multiple 3D tissues and applied two forms of mechanical stimulations (i.e., via flow or stretching) inside bioreactors for inducing EV secretions from those tissues. Under mechanical force stimulations, the EV secretions were greatly enhanced in a process mediated by Yes-associated protein (YAP) mechanosensitivity. Additionally, EVs derived from mechanically stimulated tissues containing DPSCs were more potent in inducing axonal sprouting of cultured neurons than those without mechanical stimulations, suggesting the potential of those mechanically inspired EVs for the treatment of nerve injuries, including SCI and stroke [141]. Another challenge that hampers the clinical translation of EV is the lack of a standardized isolation and purification method. So far, differential centrifugation remains the most commonly used EV separation and concentration method [102]. Other techniques include density gradients, precipitation, filtration, size exclusion chromatography and immunoisolation. Separating non-vesicular entities from EVs has not been fully achieved, and none of the current methods have both high recovery and specificity of the isolated EVs. Finally, other challenges include choosing and characterizing an appropriate cell source for EV production and standardizing potency assays and quality control criteria.

## 6. Conclusions

To conclude, the use of cells and tissue-engineered constructs provides promising therapeutic strategies for SCI, while EV-based therapy has emerged as an exciting and attractive treatment modality to orchestrate regenerative effects. Yet, despite tremendous encouraging findings of all these treatment options, key aspects such as safety, scale-up, GMP manufacturing and quality control should be considered in clinical translation. Until now, no randomized clinical trial has demonstrated the efficacy of a treatment approach for promoting functional recovery in SCI individuals. It is important to highlight that due to the complex pathophysiology of SCI, one single approach is unlikely to surmount the multifaceted hurdles. Apart from the biological interventions, other engineering strategies, such as electrical neuromodulation and activity-based rehabilitation therapy, shall be combined to win the fight against paralysis [142].

## Figures and Tables

**Table 1:** List of publications on EVs’ therapeutic effect in SCI. Abbreviations: BM: bone marrow; UC: umbilical cord; MSC: mesenchymal stem/stromal cells; NSC: neural stem cells; iv: intravenous; it: intrathecal; in: intranasal; BSCB: blood–spinal cord barrier. ↑ Score: Increased mean BBB or BMS locomotor score from EVs treatment groups, compared to untreated controls.

EVs Sources	Routes	Models	↑ Score		Proposed Mechanisms
Rat BM-MSC	iv	contusion	4.5	at wk4	anti-apoptosis, anti-inflammation, pro-angiogenesis [117]
Rat BM-MSC	iv	contusion	4.5	at wk4	neuroprotection, reduce A1 astrocytes, anti-inflammation, anti-apoptosis [118]
Rat BM-MSC	iv	contusion	3	at wk4	anti-microglia and A1 neurotoxic reactive astrocytes, anti-inflammation, anti-apoptosis, reduce scar, pro-angiogenesis [119]
Rat BM-MSC	iv	hemisection	3	at wk4	inhibit complement activation [120]
Rat BM-MSC	iv	contusion	2	at wk4	anti-apoptosis [121]
Rat BM-MSC	iv	hemisection	6	at wk4	anti-apoptosis [122]
Rat BM-MSC	iv	contusion	6	at wk4	inhibit pericyte migration, decrease BSCB permeability [123]
Rat BM-MSC (miR-133b-enriched)	iv	compression	3	at wk2	decrease RhoA expression, axon growth [115]
Rat BM-MSC (miR-29b-enriched)	iv	contusion	11	at wk8	Neuroprotection [124]
Rat BM-MSC (miR-126-enriched)	iv	contusion	7	at wk4	anti-apoptosis, pro-neurogenesis, pro-angiogenesis [116]
Rat BM-MSC (hypoxic)	iv	contusion	4	at wk4	macrophage polarization [125]
Human BM-MSC	iv	contusion	3	at wk2	decreases reactive microglia and astrocytes [126]
Human UC-MSC	iv	contusion	2	at wk8	macrophage polarization, anti-inflammation [127]
Human adipose-MSC	iv	contusion	5	at wk4	attenuate NLRP3 inflammasome activation [128]
Rat NSC	it	compression	6	at wk4	anti-inflammation [129]
Rat NSC	it	contusion	7	at wk4	inhibit NLRP3 inflammasome complex formation [130]
Rat NSC (14-3-3t-enriched)	iv	contusion	4	at wk4	enhance autophagy, anti-apoptosis, anti-inflammation [131]
Rat-NSC (IFG-1-stimulated)	iv	contusion	5	at wk4	anti-apoptosis, anti-inflammation [132]
Mouse NSC	iv	contusion	4	at wk4	activate autophagy, anti-apoptosis, anti-inflammation, anti-microglia [133]
Mouse pericytes	iv	contusion	3	at wk2	improve microcirculation, protect BSCB, anti-apoptosis [134]
Human BM-MSC (PTEN siRNA)	in	full transection	7.5	at wk8	anti-inflammation, anti-scarring, pro-angiogenesis, axon growth [35]

## Data Availability

Not applicable.
